# Cerebrovascular-Reactivity Mapping Using MRI: Considerations for Alzheimer’s Disease

**DOI:** 10.3389/fnagi.2018.00170

**Published:** 2018-06-05

**Authors:** J. J. Chen

**Affiliations:** ^1^Rotman Research Institute, Baycrest, Toronto, ON, Canada; ^2^Department of Medical Biophysics, University of Toronto, Toronto, ON, Canada

**Keywords:** cerebrovascular reactivity (CVR), Alzheimer’s disease, APOE, magnetic resonance imaging (MRI), functional MRI (fMRI), carbon dioxide (CO_2_), resting-state fMRI, mild-cognitive impairment

## Abstract

Alzheimer’s disease (AD) is associated with well-established macrostructural and cellular markers, including localized brain atrophy and deposition of amyloid. However, there is growing recognition of the link between cerebrovascular dysfunction and AD, supported by continuous experimental evidence in the animal and human literature. As a result, neuroimaging studies of AD are increasingly aiming to incorporate vascular measures, exemplified by measures of cerebrovascular reactivity (CVR). CVR is a measure that is rooted in clinical practice, and as non-invasive CVR-mapping techniques become more widely available, routine CVR mapping may open up new avenues of investigation into the development of AD. This review focuses on the use of MRI to map CVR, paying specific attention to recent developments in MRI methodology and on the emerging stimulus-free approaches to CVR mapping. It also summarizes the biological basis for the vascular contribution to AD, and provides critical perspective on the choice of CVR-mapping techniques amongst frail populations.

## Background

The brain’s energy needs are mainly met by neurovascular regulation of cerebral blood flow (CBF) (Roy and Sherrington, 1890; Duling and Berne, 1970), realized by the neurovascular unit (NVU). The NVU consists of arterial/arteriolar vascular smooth-muscle cells (VMSCs), endothelial cells, neuroglia (notably astrocytes), and pericytes. Pericytes play a crucial role in the formation and functionality of the selectively permeable space that is the blood–brain barrier (BBB), and BBB disruption is a classic marker of vascular dysfunction. Neurovascular dysfunction leads to failure to meet neuronal energy needs, which leads to oxidative stress and eventual neuronal death.

## Vascular Role In Alzheimer’s Disease

**FIGURE 1:**
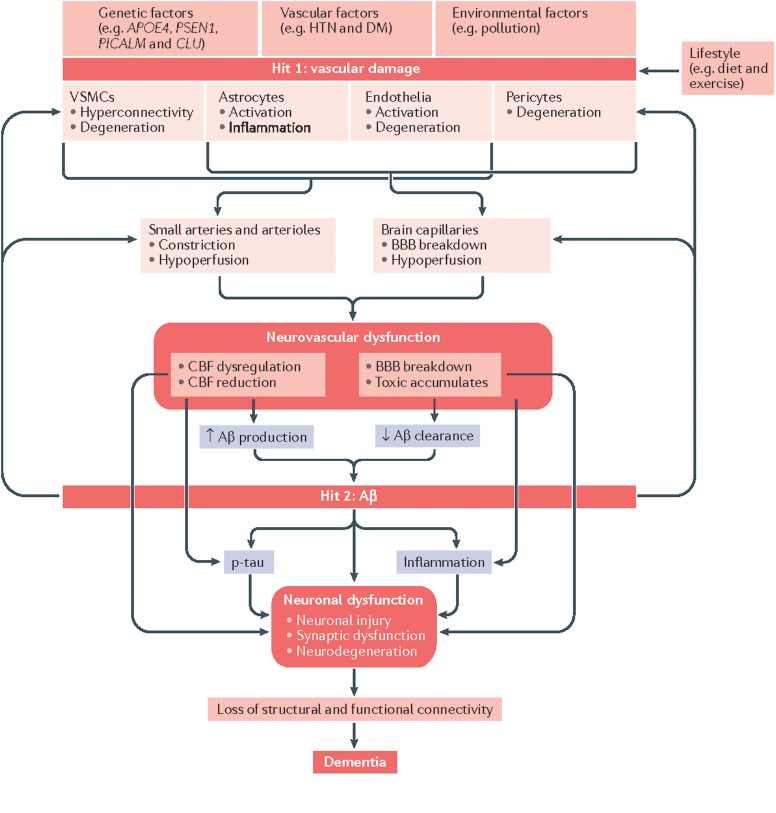
Vascular involvement in AD, as depicted in a “2-hit model” of neurovascular dysfunction. Genetic risk (including due to APOE 𝜀4) predispose individuals to amyloid deposition. Modified from Kisler et al. (2017) with permission.

While the 𝜀4 allele of the apolipoprotein E (APOE) gene is an acknowledged genetic risk factor found in 40–80% of Alzheimer’s disease (AD) patients (Strittmatter et al., 1993), and amyloid plaques are a hallmark of AD, an approximated 60–90% of AD patients also exhibit cerebrovascular pathologies (Bell and Zlokovic, 2009), supporting the vascular theory of AD. In brief, the current understanding is that genetic, environmental, and lifestyle factors may all predispose individuals to damage to the NVU (**Figure 1**). Soluble amyloid beta (Aβ), which predates Aβ plaques, deregulates cerebrovascular function by activating a free-radical cascade (Park et al., 2008), leading to compromised microvascular integrity (Dorr et al., 2012), and reduced CBF. Aβ is known to interact with endothelin-1 (Kawanabe and Nauli, 2011) and myocardin (Ramanathan et al., 2015) to promote vascular hypercontractility. Moreover, the cholinergic deficit in AD can result in a reduction of cholinergic input to cortical blood vessels (Claassen and Zhang, 2011). Furthermore, Aβ-mediated pericyte degeneration leads to BBB breakdown, increasing the perivascular accumulation of neurotoxins. The various pathways of vascular dysfunction can lead to increasing vascular tortuosity and decreasing vascular reactivity (Black et al., 2009), compromising Aβ clearance and eventually lead to neuronal death. Paradoxically, tau pathology has been associated with an increase in regional vascular reactivity (Wells et al., 2015), a controversy that is still under investigation.

Irrespective of the specific disease mechanism, vascular deficits have been demonstrated as a promising early indicator of AD. Non-invasive functional magnetic-resonance imaging (MRI) has provided strong evidence that CBF can be used to distinguish between at-risk individuals, patients and normal controls (Johnson et al., 2005). In addition, perfusion deficits have been associated with decreased functional connectivity despite maintained glucose metabolism (Göttler et al., 2018). Interventions to re-establish perfusion have been advocated as a promising preventative treatment (de la Torre, 2016). However, perfusion is a mixture of neuronal and vascular contributions, and unraveling the vascular mechanisms of AD etiology requires a more vascularly specific and routinely adoptable vascular marker. In this respect, there is early evidence that deficits in cerebrovascular reactivity (CVR) are detectable before those in CBF (Yezhuvath et al., 2012). Indeed, this was demonstrated through quantitative cerebrovascular resistance, defined as the ratio of mean-arterial blood pressure to CBF (Yew et al., 2017). Compared to CBF, resistance was found to be more sensitive at distinguishing amyloid-positive from amyloid-negative older populations as well as being more predictive of dementia conversion.

## CVR and Measurement Techniques

A recent review by Glodzik et al. (2013) provides an excellent overview of CVR measurement in AD using carbon-dioxide (CO_2_) challenges with various imaging modalities. CVR is a vasodilatory or constrictive reaction of a blood vessel to a stimulus. CVR is a well-established indicator of vascular reserve and autoregulatory efficiency. CVR decline has been associated with normal aging (Lu et al., 2011), and is the most reliable neuroimaging predictor of impending cerebrovascular disease (Pillai and Mikulis, 2015).

### Vascular Stimulus and CVR

Qualitative CVR information can be gleaned from the functional MRI (fMRI) response to any task (Dumas et al., 2012), but when quantitative CVR values are desired, vascular agents are generally required. Strong CBF responses can be induced by intravascular CO_2_ alterations, with CO_2_ inspiration thought as the optimal form of stimulus (Fierstra et al., 2013). While breathing and blood flow can both be regulated through the midbrain CO_2_ chemoreceptors, CO_2_-related blood pH changes are also actively regulated as part of maintaining homeostasis. Thus, hypercapnic challenges, in which the arterial CO_2_ content is increased, activate VMSC potassium channels (Ainslie and Duffin, 2009), leading to large CBF increases without a significant concomitant increase in metabolic rate (Chen and Pike, 2010; Jain et al., 2011). In addition, nitric oxide, which is synthesized locally following glutamate receptor activity, has also been implicated in the modulation of vasodilatory effects produced by CO_2_ (Iadecola et al., 1994).

End-tidal CO_2_ pressure (PETCO_2_) is an easily measured surrogate for arterial CO_2_ (PaCO_2_) (Battisti-Charbonney et al., 2011). PETCO_2_ is measured as the peak expired CO_2_, typically 35–40 mmHg in healthy individuals, and directly reflects alveolar CO_2_. CBF increases by 3–4% per mmHg increase in PETCO_2_, reaching its highest level when PETCO_2_ is elevated by 10–20 mmHg above normal resting value (Brugniaux et al., 2007). PETCO_2_ reductions result in CBF decline by approximately 3% per 1 mmHg change (Ito et al., 2005).

### CO_2_-Based CVR Mapping Using MRI: Methods

The clinical utility of CO_2_-based CVR quantification was established using transcranial Doppler ultrasound (TCD) (Ainslie and Duffin, 2009), positron-emission tomography (Ito et al., 2001) and dynamic X-ray computed tomography (Chen et al., 2006). While fMRI is not the most established method of assessing CVR, it offers marked advantages including richer spatial information and minimal invasiveness (Iannetti and Wise, 2007). CVR has been reliably assessed using CO_2_ fMRI in both gray and white matter (Thomas et al., 2014). In the absence of a CO_2_ delivery apparatus, breathing challenges such as breath-holding (Bright and Murphy, 2013; Pinto et al., 2016) and cued deep breathing (Bright et al., 2009) have been proposed as alternative ways to modulate intravascular CO_2_ (see **Table 1**). A comparison of breath-holding and inhaled-CO_2_ approaches reveals important CVR dependence on methodology (Tancredi and Hoge, 2013), but the reproducibility of both approaches has been established in healthy young controls (Kassner et al., 2010; Bright and Murphy, 2013).

**Table 1:** Strengths and weaknesses of various CO_2_-based CVR protocols.

Approach	Strengths	Weaknesses
End-tidal forcing	• Accurate targeting of PETCO_2_ • Can be used to produce complex PETCO_2_ shapes • Produces quantitative CVR	• Requires complex instrumentation • Feedback mechanism reduces response speed • The hypercapnic challenge may induce discomfort
Prospective targeting	• Accurate targeting of PETCO_2_ • Feed-forward mechanism enhances response speed • Can be used to produce complex PETCO_2_ shapes • Produces quantitative CVR	• Requires complex instrumentation • Requires estimation of VO2max • The hypercapnic challenge may induce discomfort
Manually blended gases	• Requires simple instrumentation and set up • Produces quantitative CVR	• PETCO_2_ response rate depends on ventilation level and cannot be controlled • PETCO_2_ not actively targeted, so resulting challenge may vary by individual
Breath-holding	• Requires no additional instrumentation	• Actual PETCO2 cannot be measured, so CVR not quantitative • Relationship between breath-hold and PETCO_2_ depends on numerous factors • Requires active subject cooperation, may vary
Cued deep breathing	• Requires no additional instrumentation	• Actual PETCO2 cannot be measured, so CVR not quantitative • Relationship between deep breathing and PETCO_2_ depends on numerous factors • Requires active subject cooperation, may vary
Resting state	• Requires little to no additional instrumentation • Requires minimal subject cooperation • Does not induce discomfort • CVR estimated from multiple PETCO_2_ values instead of block averages	• MRI response to CO_2_ is more sensitive to contamination by motion and other artifacts, given the low PETCO_2_ fluctuation amplitude

CO_2_-based CVR measured using fMRI has been widely applied and extensively cross-validated (Herzig et al., 2008). Robust hypercapnia can be induced through manually adjusted administration of blended gases (Cohen et al., 2004), end-tidal forcing (Poulin et al., 1996) or more recently, computerized PETCO_2_ targeting (Slessarev et al., 2007; Mark et al., 2010). The latter method entails the most lengthy set up but also provides immediate and robust PETCO_2_ suppression (hypocapnia) (Blockley et al., 2011), and has been proposed as part of a rapid CVR-mapping protocol for routine use (Blockley et al., 2011, 2017).

In the clinical realm, the main considerations in choosing a CVR-mapping methodology are: (1) How to assess CVR in the most non-invasive manner? (2) How to interpret the CVR information?

### Consideration for Non-invasiveness

As one of the earliest ways to induce PETCO_2_ elevation (Ratnatunga and Adiseshiah, 1990), breath-holding typically does not allow the calculation of quantitative CVR, as all participants are assumed to perform breath-holds in similar manners and the actual PETCO_2_ cannot be monitored during the challenge. The lack of PETCO_2_ monitoring is particularly concerning, as the actual change in PETCO_2_ achieved by a breath-hold depends on multiple factors, including the resting metabolic rate of the subject, lung size, recent ventilation history and whether the breath-hold is post-inspiration or post-expiration. Moreover, as typical breath-holds last 15–20 s, there are reports of poor subject compliance (Jahanian et al., 2017), particularly when elderly participants are involved. Despite these drawbacks, breath-holding-based CVR mapping has a key advantage of requiring the least instrumentation, thus allowing it to be implemented in almost any MRI scan session. Ongoing research aims to improve the robustness of breath-hold CVR mapping (Bright and Murphy, 2013), although clinical validation remains far from extensive.

Even less invasive than breath-holding, resting-state fMRI has offered a unique window to glean CVR information. Notably, Kannurpatti et al. (2014) reported a comparison of the resting-state fMRI fluctuation amplitude (voxel-wise temporal standard-deviation) as a CVR surrogate. This type of “unconstrained” or “task-free” CVR protocol does not require cooperation from participants, and is thus a promising direction of research that will likely attract tremendous attention from clinical studies. This topic will be further discussed as part of a proposed future trend.

### Consideration for Data Interpretation

Currently, the de-facto standard protocol to quantitative CVR mapping with MRI remains CO_2_ inhalation, notably controlled using computerized targeting (Kassner et al., 2010; Fierstra et al., 2013; Sobczyk et al., 2014, 2015; Poublanc et al., 2015; Sam et al., 2016; Fisher et al., 2017). Despite the complex set up, this approach has been extensively used and validated clinically. The use of modern breathing circuits also allows the CO_2_ challenge to follow nearly any shape. However, there has yet to be a consensus as to the level, duration and pattern of PETCO_2_ perturbation. As different stimulus designs likely have different vaso-stimulating capacities and hence may reveal different CVR patterns, the choice of challenge will be critical, not only in comparing across studies but also across the same individuals over time (Fierstra et al., 2013).

**FIGURE 2:**
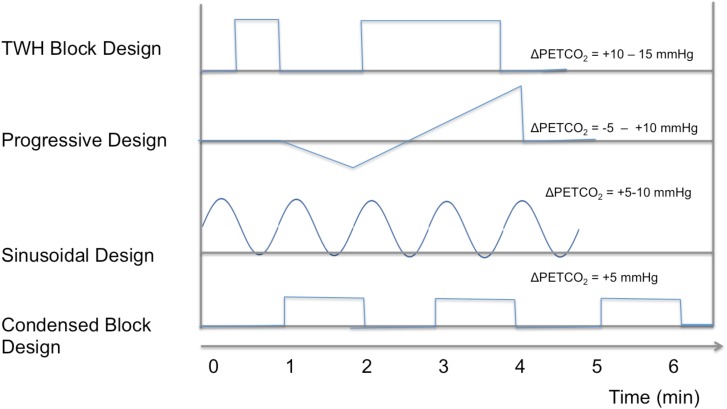
A summary of designs for various PETCO_2_ challenges in the literature.

Based on prospective targeting of stepwise PETCO_2_ changes, researchers at Toronto Western Hospital (TWH) pioneered the use of an uneven task design – one short block followed by longer block (Spano et al., 2013), both typically elevating PETCO_2_ by 10 mmHg. This design is motivated by the desire to derive more accurate estimates of CVR response time, and (Duffin et al., 2015; Poublanc et al., 2015), which may reflect regional arterial-transit time. Additionally, the same group proposed the use of progressive hypercapnia (CO_2_ ramps) (Fisher et al., 2017), in which both hypercapnia and hypocapnia are progressively induced through a ramp stimulus. It has been demonstrated that different segments of the ramp, which resulted in PETCO_2_ values of 30–50 mmHg, reveal different spatial patterns in CVR that could complement the conventional CVR information (Fisher et al., 2017). Alternatively, the use of a sinusoidal pattern allows direct estimation of response delay (as the phase in the corresponding sinusoidal CVR response), and has allowed the development of a CVR protocol as short as 5 min (Blockley et al., 2017). Such a design makes use of both hypercapnia and hypocapnia for CVR estimation, rendering estimates more robust against biases due to basal vascular tone (Halani et al., 2015). Further reducing scan time is a 1-min blended-gas protocol with 5% CO_2_ (Yezhuvath et al., 2009; Blockley et al., 2017), which has compared favorably against longer designs. These stimulation designs are summarized in **Figure 2**, and research is ongoing to validate the unique utility of each design, and it is likely that CVR measurements produced by these various methods are not directly comparable.

Concurrently, the emergence of arterial-spin labeling (ASL) MRI for CBF-based CVR mapping has added a new dimension to the choice of methods. In particular, CBF-based maps, while lower in signal-to-noise ratios (SNRs), can in fact provide more vascular-driven and thus less biased CVR quantification than BOLD fMRI (Halani et al., 2015), as demonstrated by comparisons with TCD (Gao et al., 2013). Great strides have been made in extending the use of ASL-based CVR mapping into aging research (Leoni et al., 2017), and ASL is now ubiquitously used in the study of AD (Alsop et al., 2014).

## AD-Associated Findings in Human CVR Mapping Using MRI

Cerebrovascular reactivity compromises in the middle-cerebral artery in AD, mainly measured using blended-CO_2_ method, is a well-established TCD-based finding (Lee et al., 2007; Sabayan et al., 2012; Viticchi et al., 2012; Hajjar et al., 2015). While the use of MRI-based CVR mapping in AD is still limited, its adoption is on the cusp of expansion due to rapid methodological developments.

**FIGURE 3:**
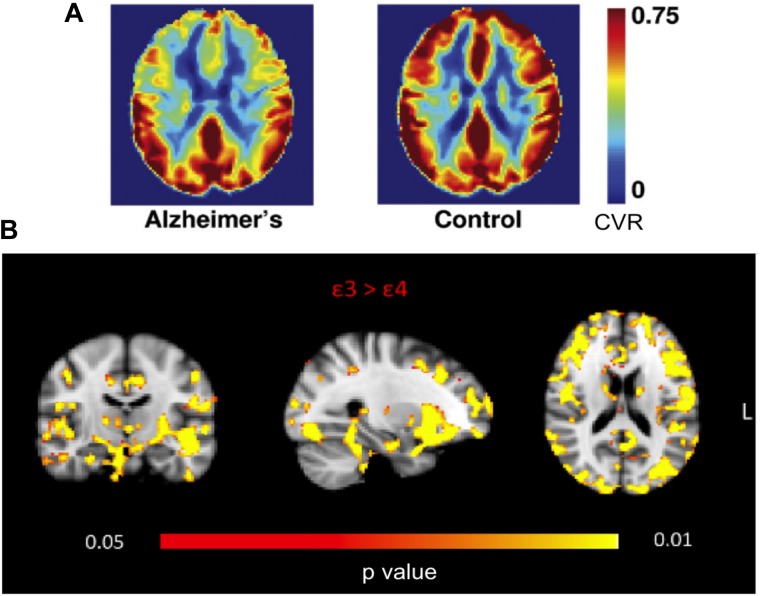
MRI-based CVR maps in AD and APOE gene carriers. **(A)** Frontal, cingular, and insular CVR deficits are found in AD patients. Figure is modified from Yezhuvath et al. (2012) with permission. **(B)** Young 𝜀4 carriers manifest widespread CVR deficits compared to 𝜀3 homozygotes. Figure is modified from Suri et al. (2014) with permission.

Using MRI, such CVR reductions have been localized to the prefrontal, anterior cingulate and insular regions (Yezhuvath et al., 2012). Interestingly, while this pattern overlapped little with that of CBF deficits (found in the temporal and parietal regions), it agreed with the localization of amyloid deposition (Yezhuvath et al., 2012), suggesting that CVR has unique sensitivity to AD pathology (**Figure 3A**). Moreover, cortical and white-matter CVR deficits have been linked to the incidence of leukoaraiosis (Yezhuvath et al., 2012; Sam et al., 2016). Such reductions in CVR echo postmortem observations of vascular dysfunction (Chow et al., 2007), and can be the result of a number of structural changes in the vasculature, including cerebral amyloid angiopathy (CAA), astrocytic end-feet swelling, pericyte degeneration, basement-membrane hypertrophy and endothelial-cell metabolic abnormalities (Hashimura et al., 1991; Miyakawa et al., 1997).

Cerebrovascular reactivity deficits have been discovered amongst young APOE 𝜀4 gene carriers (Hajjar et al., 2015), even when compared to 𝜀3 homozygotes (Suri et al., 2014) (**Figure 3B**). Such deficits are found to be widespread, notably in the prefrontal and parahippocampal regions, bolstering the hypothesis that genetic predisposition to vascular disease contributes to the vulnerability of 𝜀4-carriers to late-life pathology (Kisler et al., 2017).

It is increasingly recognized that vascular deficits may be the most accessible physiological treatment target in the effort to delay dementia onset, and approaches that enhance perfusion have demonstrated potential therapeutic value (de la Torre, 2016). Predicting progression of preclinical AD amongst mild-cognitive impaired (MCI) individuals has been a key research focus. Using breath-hold TCD, the predictive value of CVR (Sato and Morishita, 2013) in terms of MCI-to-AD conversions has been demonstrated (Buratti et al., 2015).

In light of the overwhelming influence of vascular risk factors in AD progression, the lines between vascular deficits in AD and other types of dementia can become blurred in later stages of the disease, as will be discussed in later sections. As a case in point, given the rampant occurrence of CAA amongst suspected AD patients, the vascular dysfunction can produce deleterious oxidative stress that can promote ischemia and accelerate AD progression (Girouard and Iadecola, 2006; Bookheimer and Burggren, 2009). Furthermore, CVR may be a more sensitive early marker of AD severity (Yezhuvath et al., 2012). It is conceivable that a diseased vasculature may sustain normal perfusion but reveal an abnormal response to a stress test such as used in CVR mapping (Fierstra et al., 2013). Nonetheless, as an increasing amount of CVR data is generated using BOLD fMRI, it is also important to note that microvascular CVR is more reflective of AD severity (Jellinger and Attems, 2006), while the BOLD fMRI signal is generally dominated by large-vessels. This is true at clinical field strengths (1.5 or 3 Tesla) and using either gradient- or spin-echo BOLD. ASL, on the other hand, is likely more sensitive to capillaries and arterioles, and should be the most natural alternative for CVR mapping.

There are numerous fMRI studies that report age-related differences in the BOLD response amplitude or extent, but as the BOLD response to neuronal stimuli is intrinsically modulated by CVR, one must be cautioned against interpreting age-related BOLD differences as neuronal differences. This is also true of resting-state fMRI, where functional connectivity has been found to vary with CVR (Golestani et al., 2016a; Lajoie et al., 2017; Chu et al., 2018).

## Research Gaps and Emerging Topics

As stated earlier, the most commonly reported challenge in acquiring CVR maps in clinical research pertains to the need for subject cooperation. This is true for all of the stimulus designs described thus far, imposing a fundamental limitation on the routine use of CVR mapping amongst patients. Very recently, resting-state methods that do not require CO_2_ perturbation have flourished (Golestani et al., 2016b; Jahanian et al., 2017; Liu et al., 2017). Resting-state CVR methods rely on intrinsic fluctuations in the BOLD fMRI signal, and may significantly broaden the accessibility of CVR mapping to clinical researchers. Additionally, beyond the magnitude of CVR, the dynamic features of the fMRI response can also provide useful information. A slowing of the CVR response has been shown to characterize vascular lesions (Poublanc et al., 2015), adding a dimension to the utility of CVR mapping.

The response of the cerebral circulation to a changing arterial CO_2_ concentration is not linear – the circulatory response follows a sigmoidal shape, and is greater for hypercapnia than to hypocapnia (Ogoh et al., 2008; Peebles et al., 2008; Rodell et al., 2012). Moreover, it is critical to note that while CVR is traditionally defined as a blood-flow response (as is the case in TCD, PET, and CT), the BOLD signal is not a direct measure of CBF. Rather, BOLD is modulated by CBF, CBV, and baseline oxidative metabolism, not to mention a series of field-dependent physical variables. Thus, the assumption of a linear relationship between the BOLD and CBF responses to CO_2_ is likely tenuous. Specifically, it is widely known that the BOLD response varies with CBF in a non-linear fashion (Hoge et al., 1999). This non-linearity is superimposed in the inherently sigmoidal vascular response to CO_2_ (Battisti-Charbonney et al., 2011). Such non-linear CVR changes have been demonstrated through a comparison with CBF-based CVR measurements at various vascular baselines (Halani et al., 2015), and may in a small part underlie the BOLD response behavior in the “vascular steal” phenomenon (Sobczyk et al., 2014). This limitation will require careful consideration in the presence of known vascular dysfunction (Battisti-Charbonney et al., 2011).

A critical assumption for CVR mapping is that PETCO_2_ represents PaCO_2_. However, PaCO_2_ is determined by both inhaled CO_2_ and the minute ventilation. Low cardiac output can increase alveolar dead space, which would increase the difference between PaCO_2_ and PETCO_2_ (Shibutani et al., 1992), leading to underestimations of PETCO_2_-based CVR. In addition, PETCO_2_ is shown to overestimate PaCO_2_ during exercise in young adults, but not in older adults (Williams and Babb, 1997). Moreover, PETCO_2_-related CVR is known to follow a circadian rhythm, increasing with the level of alertness (Ainslie and Duffin, 2009). These factor contribute to inter-cohort, inter-sessional and inter-subject variability in CVR estimates that must be accounted for when assessing true differences in CVR.

In this regard, an emerging research direction is building normative CVR atlases that allow the significance of CVR deviations to be assessed (Sobczyk et al., 2015). Such atlases would ideally encompass not only quantitative CVR values but also CVR-timing information (van Niftrik et al., 2017). This is a critical step in expanding the clinical utility of CVR maps, and atlases will likely need to be specific to the CO_2_ delivery method, stimulation design, study objectives and MRI system used.

The above research gaps pertain not only to AD but to other cerebrovascular diseases also. The increasing awareness of the vascular etiology of various forms of dementia will highlight these limitations and prompt more focused validation studies.

## Author Contributions

The author confirms being the sole contributor of this work and approved it for publication.

## Conflict of Interest Statement

The author declares that the research was conducted in the absence of any commercial or financial relationships that could be construed as a potential conflict of interest.
